# Cat scratch disease in a 23-year-old male–Case report

**DOI:** 10.3389/fpubh.2022.1046666

**Published:** 2023-01-13

**Authors:** Radeyah Waseem, Muskan Seher, Sohiba Ghazal, Hussain Haider Shah, Ume Habiba

**Affiliations:** Dow University of Health Sciences, Karachi, Sindh, Pakistan

**Keywords:** cat scratch disease, *Bartonella henselae*, fever, lymphadenopathy, infection

## Abstract

Cat-scratch disease (CSD) is an infectious disease that usually presents with fever, headache, loss of appetite, weight loss, tender lymphadenopathy, and other symptoms. CSD is also the most common cause of infectious lymphadenitis in children, adolescents, and young adults. This contagious disease most often results from a scratch or bite of a cat. The course of this disease depends on the patient's immune status. CSD sometimes presents as a systemic disease and leads to various disease entities. In this study, we describe the case of a 23-year-old man exhibiting fever, generalized weakness, and neck swelling. The patient was unconscious when presented to the Emergency Department. He was given at least 3–4 liters IV bolus of 0.9% normal saline, but it failed to raise the blood pressure. He was then given an inotropic drug (noradrenaline) for low blood pressure and antibiotics (azithromycin) for fever. Venereal disease research laboratory (VDRL) and human immunodeficiency virus (HIV) serology came out negative. Histopathology ruled out tuberculosis and malignancy and confirmed necrotizing/suppurative granulomatous inflammation. These features favor the diagnosis of CSD.

## Background

Principally caused by the Gram-negative rod *Bartonella henselae*, cat-scratch disease (CSD) is one of the most common causes of chronic lymphadenopathy in adolescents and children. It has obscure international incidence despite cases being reported globally, and seropositive prevalence rates show wide fluctuations between 0.6 and 37%. An association with warm, humid climates has been established (1). As depicted in a study conducted by Nelson et al. (2) spanning the time period between 2005 and 2013, the incidence peaked in 2005 (5.7/100,000 population) and gradually tapered to 4.0/100,000 in 2013 (2).

While CSD classically comes to clinical attention with a distinct inoculum site and regional lymphadenopathy, 10% of patients can present with atypical features which embody multi-system involvement. These include the cardiovascular system, musculoskeletal system, and neurological, dermal, and other regional sites, coupled with bacteremia. A prototypical case manifesting these findings has been reported in a previous case report by De Keukeleire et al. (3).

Cat-scratch disease has been barely reported in Pakistan, with the first documented case being reported in Rawalpindi in 2018 and published in 2020 (4). A new case has now been found in Karachi on 29 May 2022.

## Case presentation

**Figure 1:**
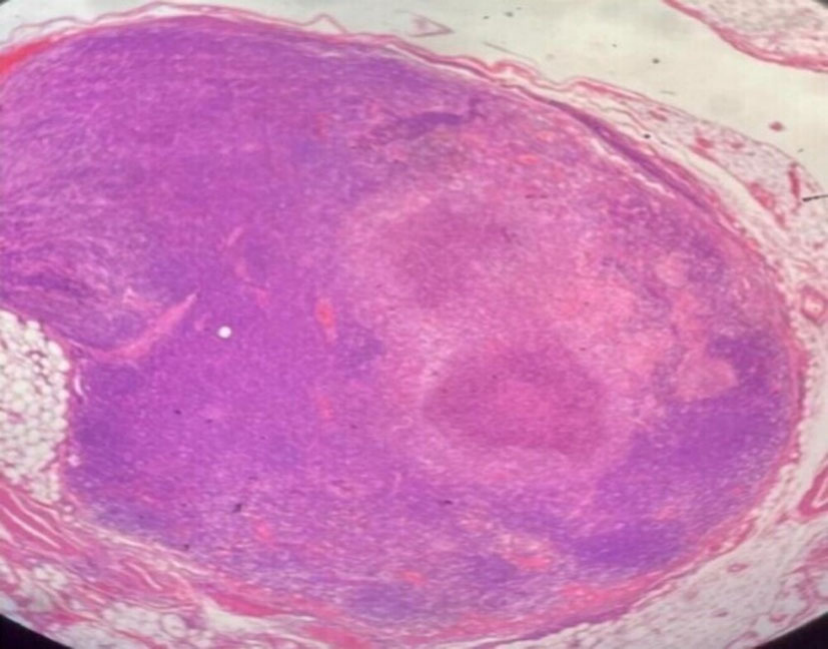
Section examined reveals lymph node showing areas of necrosis and abscess surrounded by palisading histiocytes along with capsular and subcapsular granulomas.

A 23-year-old man with no known co-morbidities presented in the Emergency Department at a Tertiary Care Hospital in Karachi with multiple complaints of neck swelling for 1 year, fever for 1 month, and generalized weakness for 1 week. He was a resident of Khuzdar, unmarried, and a final-year student of Doctor of Veterinary Medicine. On detailed history, the single neck swelling presented anteriorly and was not associated with any joint swelling, morning stiffness, or tenderness in small joints. It also regressed with time. However, he then noticed another static swelling on the right side of the neck in close proximity to the previous one. Fever was reported as intermittent and documented up to 104°F. It was associated with rigors and chills but subsided after oral paracetamol. It was associated with severe body aches and joint pain involving the wrists, elbows, and knees bilaterally. No diurnal variation or night sweats were accounted for, nor was any rash or oral ulcer. There was no history of Raynaud's phenomenon, burning micturition, cough, or loose stools. On further inquiry, it was revealed that he had a pet cat for a duration of 1 year in 2020 and is a student of Doctor of Veterinary Medicine (DVM), reports positive for exposure to various animals. His personal history revealed no addictions, but there was a history of sexual contact. On examination, three to four swellings were accounted for in the right posterior cervical region, the largest one having dimensions of ~2.0 × 1.0 cm. They were non-tender, non-erythematous, firm, and immobile, with no discharging sinus. Two small swellings were also noted bilaterally in the inguinal region. These were also found to be non-tender, non-erythematous, firm, and immobile with no discharging sinus. They measured ~1.0 × 1.0 cm. Blood pressure was recorded as 80/40 mm Hg, pulse 92 beats/min, oxygen saturation 98% on room air, and temperature as afebrile in the Emergency Department. On systemic examination, the abdomen was found to be soft and non-tender, with no visceromegaly noted; the chest, cardiovascular system, and central nervous system were also found to be unremarkable. The hospital course was initiated by giving IV fluids and commencing ionotropic drugs (noradrenaline) to raise his blood pressure, and he was hospitalized. Meropenem 1 g was started IV three times a day (TDS) secondary to high total leukocyte count (TLC = 49) and procalcitonin (PCT = 8.17). As the diagnosis was uncertain and the patient presented with fever, to rule out infective endocarditis and vegetation, echocardiography was scheduled, which was found to be unremarkable. Next, the general surgery team was consulted for an excisional biopsy of the right cervical lymph node. He was then stepped down to a private room as blood pressure started to show improvement, and ionotropic support was eventually tapered off. Azithromycin 500 mg was added to the treatment regime as there was no improvement in fever. The Infectious Diseases Department next advised VDRL and HIV serology (which came out to be negative), and azithromycin was switched to an injection of ciprofloxacin 500 mg IV twice daily. Histopathology ruled out tuberculosis and malignancy, and as fever started to space out, meropenem and ciprofloxacin were continued. After about 5–6 days, the histopathology department came up with the diagnosis of cat-scratch disease. The patient was fever-free for 2 days, clinically and vitally stable, and discharged on doxycycline 100 mg and rifampicin 300 mg twice daily for 6 weeks. Figure 1 shows a section of a lymph node showing areas of necrosis and abscess surrounded by palisading histiocytes along with capsular and subcapsular granulomas. Table 1 highlights results of important laboratory investigations to reach a diagnosis.

**Table 1:** Results of laboratory investigations.

**Laboratory investigation**	**Results**	**Normal reference range**
**Hematology**		
Hemoglobin (gm/dl)	13	13–18
TLC (per liter)	49.7	4–10 × 10^9^
PCV (%)	39.7	36–54
Platelet count (× 10^9^ /L)	235	150–400 × 10^9^
PT (seconds)	12	11–13.5
APTT (seconds)	22	21–35
INR	1.21	0.8–1.1
**Renal profile**		
Urea (mg%)	35	10–50
Creatinine (mg%)	1.15	0.5–1.5
Chloride (mmol/L)	105	0–111
Potassium (mmol/L)	2.7	3.5–5.3
Sodium (mmol/L)	138	137–150
Bicarbonate (mmol/L)	21	22–34
Magnesium (mg%)	1–2.9	1.9–2.5
Calcium (mg%)	7–8.3	8.1–10.4
Phosphorus (mg%)	3.84	2.5–1.5
**Liver profile**		
Bilirubin total (mg%)	0.68	0.2–1.2
Bilirubin direct (mg%)	0.40	0.0–0.25
Bilirubin indirect	0.28	
SGPT/ALT (Units/L)	65	Upto 40
Alkaline phosphatase (Units/L)	54	117
Gamma-GT (Units/L)	64	7–22
Amylase (Units/L)	75	28–100
Lipase (Units/L)	35	0–160

TLC, Total Leucocyte Count; PCV, Packed Cell Volume; PT, Prothrombin Time; APTT, Activated Partial Thromboplastin Time; INR, International Normalized Ratio.

## Discussion

CSD is a feline infection that is transmitted to humans by a scratch, bite, or contamination of a wound or mucous membrane, especially by young cats (kittens) (5). It is also referred to as cat-scratch fever or subacute regional lymphadenitis. The disease commonly resolves spontaneously in the majority of immunocompetent patients; however, rare cases have been identified where it may progress to present atypical, systemic features, such as oculoglandular syndrome, neuroretinitis, pneumonia, arthralgia, arthritis, encephalitis, and thrombotic thrombocytopenic purpura (6). Hepatic and splenic involvement is occasionally reported and warrants investigation to exclude more conventional causes. It was reported by Rocco et al. (7) on a black, 16-year-old man who presented with disseminated signs, neck swelling, and hepatosplenomegaly (7). Involvement is in the form of granulomas that appear on ultrasound as multiple, rounded, well-circumscribed, hypoechoeic lesions with diameters ranging from 0.5 to 3 cm (8). Probable diagnosis comprises an exhaustible list of bacterial diseases inclusive of *M. tuberculosis, M. avium, F. tularensis, T. whippelii, C. burnetti, L. monocytogenes*, and *C. psittaci*, among others (9). Infection in an immunocompetent host is usually self-relenting, resolving within a few weeks. The initial stage of infection presents as papules or pustules at the site of the inoculum, and erythema nodusum accompanied by prodromal features such as fever and fatigue. Regional lymphadenopathy concurrently develops, characteristically unilaterally and involving a solitary node in half of the population reporting, with the other half progressing to involve multiple nodes or discrete regions. CSD may also disseminate systemically, with eyes being most frequently involved. Eye involvement may report clinically with a plethora of presentations, including neuroretinitis, anterior uveitis, choroiditis, Parinaud syndrome, retinal vessels occlusion, and inflammatory disc edema, among others (10, 11). The disease may also present atypically in immunocompetent patients precipitating deleterious consequences including cardiac complications (endocarditis), respiratory manifestation (pneumonia and pleural effusion), musculoskeletal involvement (osteomyelitis and paravertebral abscess), and CNS involvement (aseptic meningitis and encephalopathy) (12). Infection in an immunocompromised host follows a very different course, manifesting as bacillary angiomatosis–peliosis, which is symbolized by angioproliferative lesions mimicking those of Kaposi sarcoma in various sites amalgamating the skin, the liver, the spleen, the bone, and various others (1). The most notable causative agents of the disease are the *Bartonella* species, namely *Bartonella henselae, Bartonella quintana, Bartonella elizabethae*, and *Bartonella vinsonii*. *B. henselae* and *B. quintana* are habitually associated with human disease, while *B. elizabethae* infrequently harbors infection in immunocompromised patients. No cases have been reported of *B. vinsonii* in humans (13). In some cases, treatment with antibiotics such as azithromycin can be helpful. Other standard antibiotics used for treating CSD include clarithromycin, rifampin, trimethoprim-sulfamethoxazole, and ciprofloxacin. CSD classically follows a variable clinical course, initially presenting at the site of inoculation, extending to involve multiple lymph nodes, and finally presenting general, systemic symptoms. It commences as lone or multiple erythematous papules 3–4 days after inoculation at the site of infection, and a detailed history is vital to a proper diagnosis. The papule undergoes different stages from erythematous to vesicular to papular and finally to the crusted stage. Typically, this is followed by regional lymphadenopathy 1–3 weeks after the initiating cause and frequently involves a single node, relapsing after several months. Axillary and epitrochlear nodes and lymph nodes of the head, neck, and groin may also be involved asymmetrically. The nodes are found to be hyperechoic, multiple, and highly vascularised when visualized under an ultrasound examination (14). Approximately half of the reported cases may develop mild systemic symptoms constituting abdominal and generalized pain, malaise, and anorexia (15). Histopathological examination of the lymph nodes reveals a granulomatous picture alongside microabscesses and focal necrosis (16). Atypical involvement of CSD involves hepatosplenic CSD, cardiopulmonary CSD, CSD-associated osteomyelitis, central nervous system involvement, and systemic and bacteremic CSD. The diagnosis of CSD is conventionally made when three of four criteria are met: (1) positive history of contact supported with a primary inoculation site, (2) a supporting CSD skin test result, (3) exclusion of other potential causes of subacute lymphadenopathy, and (4) biopsy results showing the specific histopathological features (13).

## Conclusion

With the second case of CSD being documented in the country, it is crucial that a proper diagnosis is made as the clinical picture of the disease imitates various chronic illnesses like tuberculosis or malignancy. Pakistan is endemic to tuberculosis which has profound similarities in regard to the clinical presentation of CSD, emphasizing the need for a proper diagnosis. The differential diagnosis of a patient presenting with lymphadenopathy should consider cat-scratch disease a potential cause, especially in children. Due to its uncommon incidence in Pakistan, clinicians rarely consider CSD a possible cause of a patient's symptoms. However, the recent reporting of two cases in different provinces (Punjab and Sindh) and age groups (8 and 23 years) highlights the importance of being cognizant of the possibility of the disease whenever a patient presents with symptoms like lymphadenopathy syndrome, undifferentiated arthritis, and a fever with no underlying cause. Immaculate history and examination need to be conducted, and relevant expertise must be sought to make the proper diagnosis and treat the patient accordingly to prevent iatrogenic compromise in the quality of life of the afflicted.

## Data availability statement

The raw data supporting the conclusions of this article will be made available by the authors, without undue reservation.

## Ethics statement

Ethical review and approval was not required for the study on human participants in accordance with the local legislation and institutional requirements. The patients/participants provided their written informed consent to participate in this study. Written informed consent was obtained from the individual(s) for the publication of any potentially identifiable images or data included in this article.

## Author contributions

RW and MS: conceptualization and writing—original draft. SG: writing—original draft. HS and UH: writing—review and editing. All authors contributed to the article and approved the submitted version.
